# Naringin Attenuates the Diabetic Neuropathy in STZ-Induced Type 2 Diabetic Wistar Rats

**DOI:** 10.3390/life12122111

**Published:** 2022-12-15

**Authors:** Md Fahim Ahmad, Nida Naseem, Inamur Rahman, Nazia Imam, Hina Younus, Swaroop Kumar Pandey, Waseem A. Siddiqui

**Affiliations:** 1 Research Lab-1, Interdisciplinary Biotechnology Unit, Aligarh Muslim University, Aligarh 202002, India; 2 Department of Life Sciences Ben-Gurion, University of the Negev, Beer Sheva 8410501, Israel

**Keywords:** diabetic neuropathy, diabetes mellitus, naringin, streptozotocin, proinflammatory cytokines, neuronal specific markers, histopathology

## Abstract

The application of traditional medicines for the treatment of diseases, including diabetic neuropathy (DN), has received great attention. The aim of this study was to investigate the ameliorative potential of naringin, a flavanone, to treat streptozotocin-induced DN in rat models. After the successful induction of diabetes, DN complications were measured by various behavioral tests after 4 weeks of post-induction of diabetes with or without treatment with naringin. Serum biochemical assays such as fasting blood glucose, HbA1c%, insulin, lipid profile, and oxidative stress parameters were determined. Proinflammatory cytokines such as TNF-α and IL-6, and neuron-specific markers such as BDNF and NGF, were also assessed. In addition, pancreatic and brain tissues were subjected to histopathology to analyze structural alterations. The diabetic rats exhibited increased paw withdrawal frequencies for the acetone drop test and decreased frequencies for the plantar test, hot plate test, and tail flick test. The diabetic rats also showed an altered level of proinflammatory cytokines and oxidative stress parameters, as well as altered levels of proinflammatory cytokines and oxidative stress parameters. Naringin treatment significantly improved these parameters and helped in restoring the normal architecture of the brain and pancreatic tissues. The findings show that naringin’s neuroprotective properties may be linked to its ability to suppress the overactivation of inflammatory molecules and mediators of oxidative stress.

## 1. Introduction

Diabetic neuropathy (DN) is the main consequence of long-term diabetes and one of the abnormalities it produces. It includes symptoms that significantly affect the patient’s quality of life and emotional well-being, such as hyperalgesia, allodynia, dysesthesia, and a burning feeling [1]. DN is associated with several disorders, including vascular [2], metabolic, and neurotrophic abnormalities [3]. These anomalies result in electrophysiological changes, aberrant sensory perception, and injury to nerve fibers [4]. Additionally, DN results in neuronal degeneration and changes to the activities of insulin-like growth factor (IGF) and nerve growth factor (NGF) [5].

Diabetes impacts the central nervous system and causes disturbances such as behavioral changes, autonomic dysfunctions, altered neuroendocrine functions, and neurotransmitter alterations and thus leads to end-organ damage [6,7]. A number of mechanisms, including polyol, hexosamine, protein kinase C, advanced glycation, poly (ADP-ribose) polymerase, oxidative stress, and inflammation, are involved in the development of DN and degeneration. DN, also known as peripheral nerve disorder or diabetic peripheral neuropathy (DPN), affects organs such as the heart, kidney, liver, eyes, etc., which are stimulated by the autonomic nervous system. More than 50% of all diabetic individuals experience DN, which is typically associated with a loss of sensation in the feet and weak foot muscles that make walking difficult [8]. The main component that triggers the activation of inflammatory and apoptotic pathways, insufficient levels of neurochemical growth factors, abnormalities of the neurovasculature, demyelination of neurons, and enhanced autoimmune damage is oxidative stress [9]. Therefore, it is an option to prevent diabetic neuropathy progression by inhibiting oxidative stress and inflammation cascades.

Plants are the primary source of medicine used for the treatment of diabetes in India and another ancient system of drugs in the world. Of late, greater attention has been given to natural phytocompounds that embed antioxidant activity in them. Hence, they are now being employed to remove reactive species for therapeutic purposes, leading to attenuation of oxidative-stress-mediated diabetes. Insulin resistance, dysfunction in beta-cell, and insulin secretion are caused by persistent oxidative stress in diabetic subjects. Phytochemicals can, however, moderate these side effects by either controlling blood sugar levels or attenuating at least one of the following insulin resistance-related mechanisms: beta-cell activity, glucose (re)absorption, and mechanisms involved in incretion [10].

One of the primary active ingredients of Chinese herbal medicines, including Drynariafortunei (Kunze) J. Sm. (D.F.), *Citrus aurantium* L. (C.A.), and *Citrus medica* L. (CM), is naringin (NR), a flavanone glycoside produced from the flavanone naringenin and the disaccharide neohesperidose [11,12]. It is also abundant in citrus fruits [13] and imparts a bitter taste to citrus juices [14]. Flavonoids, an essential group of secondary metabolites, are a source of bioactive compounds in plants [15]. Several studies have shown that naringin has been found to have antioxidant, anti-inflammatory, antiapoptotic, antiulcer, antiosteoporotic, and anticarcinogenic activities [16]. It has also been found that NR may be a strong therapeutic candidate against methotrexate-induced reprotoxic effects, renal toxicity, and antitumor efficacy in HepG2 cells [17,18,19]. This study aimed to explore the therapeutic potential of naringin for diabetic neuropathy. We carried out a series of in vivo studies to investigate the protective role of naringin to overcome behavioral changes, as well as biochemical, cytokine analysis, and histopathological examination of brain and pancreatic tissues.

## 2. Materials and Methods

### 2.1. Materials

The following substances were bought from Sigma-Aldrich (Sigma-Aldrich, St. Louis, MO, USA): trichloroacetic acid (TCA), glutathione reduced (GSH), 5,5′-dithiobis(2-nitrobenzoic acid) (DTNB), thiobarbituric acid (TBA), ethylene diamine tetraacetic acid (EDTA), 2,4-dinitrophenylhydrazine (DNPH), and streptozotocin (STZ). Kits for serum biochemical analysis and lipid profiles were purchased from Agappe Diagnostics Ltd. (Kerala, India), and kits for proinflammatory cytokines were procured from RayBiotech (RayBiotech, Peachtree Corners, GA, USA). All other chemicals and solvents utilized in this study were analytical-grade products that were readily available in stores.

### 2.2. Animals

For this investigation, adult male Wistar albino rats (*Rattus norvegicus domestica*) weighing 120–180 g were employed. The animals were housed in the animal house of Ajmal Khan Tibbiya College, of Aligarh Muslim University, Aligarh, in a controlled environment under standard conditions of temperature and humidity with an alternating 12 h light and dark cycle. Ideally, 4 animals were housed per cage. The animals had free access to food and water ad libitum. The Committee for Control and Supervision of Experiments on Animals’ (CPCSEA) rules were followed in caring for the animals.

**Figure 1:**
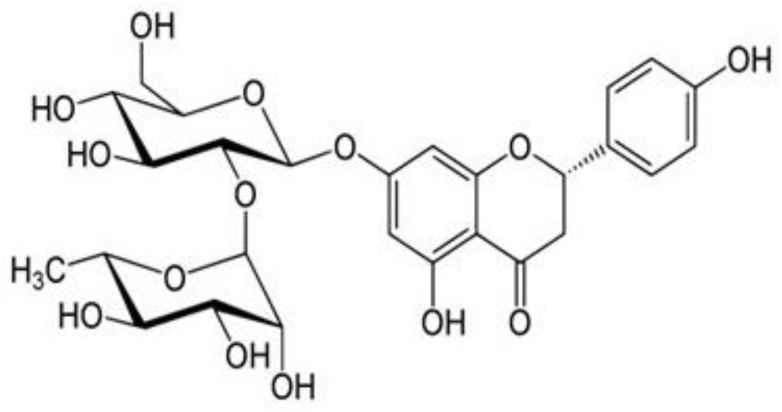
Structure of naringin.

Six groups of at least six each were formed from the animal population. Rats in Groups I, II, and III served as normal control, diabetic control, and standard, respectively, while those in Groups IV, V, and VI were fed orally with different doses of naringin (Figure 1), i.e., 10, 20, and 40 mg/kg b.w, respectively.

### 2.3. Experimental Design

Male Wistar rats of Groups II (diabetic control), III, IV, V, and VI were fed a high-fat diet (HFD), with water ad libitum for four weeks instead of standard rat chow, to make them obese, hence increasing the chances of type 2 diabetes mellitus (T2DM) induction. A single intraperitoneal injection of streptozotocin (STZ, 35 mg/kg BW) at a low dose was given at the conclusion of the four-week period in citrate buffer (pH 4.5). Fasting blood glucose levels were assessed seven days later, and animals having a fasting blood glucose level of more than or equal to 250 mg/dL were only considered for study (Figure S1).

Group I: Normal control (NC) rats received a standard chow diet throughout the experiment.

Group II: Diabetic control (DC) rats received a high-fat diet (HFD) post-injection with streptozotocin until the duration of the experiment.

Group III: Standard (Std) diabetic rats received standard antidiabetic drug (glibenclamide) at 5 mg/kg BW/day, orally for four weeks post-induction of diabetic complications.

Group IV: Diabetic rats received NR-1 (10 mg/kg BW/day) orally for four weeks post-induction of diabetic complications.

Group V: Diabetic rats received NR-2 (20 mg/kg BW/day) for four weeks post-induction of diabetic complications.

Group VI: Diabetic rats received NR-3 (40 mg/kg BW/day) for four weeks post-induction of diabetic complications.

### 2.4. Induction of Type 2 Diabetes Mellitus

Male Wistar rats of Groups II (diabetic control), III, IV, V, and VI were fed a high-fat diet (HFD), with water ad libitum for four weeks instead of standard rat chow, to make them obese, hence increasing the chances of type 2 diabetes mellitus (T2DM) induction. A single intraperitoneal injection of streptozotocin (STZ, 35 mg/kg BW) at a low dose was given at the conclusion of the four-week period in citrate buffer (pH 4.5). Fasting blood glucose levels were assessed seven days later, and animals having a fasting blood glucose level of more than or equal to 250 mg/dL were only considered for study. Hyperglycemic rats were considered to be diabetic, and further, these groups were fed an HFD for four weeks for developing complications such as diabetic neuropathy.

### 2.5. Validation of Diabetic Neuropathy Rats

Four weeks after the induction of diabetes, animals were assessed for the development of diabetic neuropathy. The following characteristics were examined for the physical validation of neuropathic pain: the acetone drop test, pinprick test, plantar test, tail flick test, and tail pinch test. After successful development of diabetic neuropathy, animals were treated with three different doses of naringin for four weeks. After four weeks, physical parameters were studied, and animals were sacrificed for further studies.

### 2.6. Sample Collection and Tissue Preparation

Under general anesthesia using isoflurane, the rats were sacrificed, and blood was drawn by puncturing their hearts. For the analysis of serum biochemistry, blood was drawn. Brain and sciatic nerves were swiftly removed and homogenized in 10% phosphate-buffered saline (pH 7.4) using a mortar and pestle, and some parts were stored in formalin for histopathological analysis. To obtain mitochondria, the homogenate was centrifuged 1000× *g* for 5 min, and resuspended in a buffer (sucrose 0.32 M, EDTA-K+ 1.0 mM, Tris–HCl 10 mM, pH 7.4), and homogenized again. The residual extract was centrifuged at 1000× *g* for 5 min. Postmitochondrial supernatant (PMS), which was obtained from homolysate after centrifuging it at 15,000× *g* for 20 min at 4 °C, was isolated for further research.

### 2.7. Assessment of Neuropathic Pain

#### 2.7.1. Acetone Drop Test

The acetone drop test was performed according to the method of Choi and workers [20]. For this procedure, 100 µL of acetone was administered to the right hind paw’s plantar surface and left there for a minute to observe how chilly it felt.

#### 2.7.2. Plantar Test

The plantar test was performed according to the procedure of Erichsen and Blackburn-Munro [21] with slight modifications. The right hind paw’s plantar surface was treated with microfilaments of different pressures in a perpendicular direction. The fast retraction of the hind leg in response to pain, which was detected, was what caused the intensity. The midplantar surface received the stimulation, just six times each minute, to prevent unintended tissue damage.

#### 2.7.3. Hot Plate Test

The hot plate test was performed according to the procedure of Hargreaves and coworkers [22] with slight modifications. This indicated that the right hind paw was on a hot plate, the temperature of which was maintained at 52 °C ± 2 °C, and the cutoff time was set to 20 s. The right hind limb was quickly removed, which was observed as a thermal hyperalgesic response.

#### 2.7.4. Tail Flick Test

This test was performed according to the method of D’Aemour and Smith [23] with some modifications. The tails of rats were subjected to immersion in a beaker filled with warm water at a temperature of 65 °C ± 5 °C. Tail withdrawal latency, a measure of mice’s temperature sensitivity, was discovered. It was thought that removing the tail from the plantar device quickly indicated the onset of neuropathic pain. The cutoff time for the experiment was set at 15 s.

### 2.8. Serum Biochemical Estimation

#### 2.8.1. Fasting Blood Glucose Measurement

The glucose-oxidase-peroxidase (GOD/POD) method was used to test fasting blood glucose using a commercial diagnostic kit from Dr. Morepen, Morepen Laboratories Ltd. (Himachal Pradesh, India). Under general anesthesia, blood was taken from the retro-orbital plexus, and fasting blood glucose (FBG) was calculated for each group both before and after medication therapy.

#### 2.8.2. Estimation of Serum Insulin Level

The RayBio Rat Insulin ELISA kit, an in vitro enzyme-linked test for the quantitative detection of rat insulin in serum, was used to quantify insulin. In this test, a 96-well plate with an antibody that is specific for insulin is used. Insulin contained in a sample was bound to the wells with the assistance of the immobilized antibody after both standards and samples were pipetted into the wells. After washing the wells, an antirat insulin antibody that had been biotinylated was added. Streptavidin that had been horseradish peroxidase (HRP)-conjugated was added to the wells after the unbound biotinylated antibody was washed away. A 3,3′5,5′-tetramethylbenzidine (TMB) substrate solution was added to the wells after a second round of washing, and color developed in direct proportion to the amount of insulin bound. The stop solution altered the color from blue to yellow, and a plate reader (Bio-Rad, iMark microplate reader) assessed the color’s intensity at 450 nm.

#### 2.8.3. Determination of Percent Glycated Hemoglobin (%HbA1c) Level

%HbA1c was assayed by the ELISA method using a diagnostic kit from Agappe Diagnostics Ltd. (Kerala, India) obeying the guidelines provided by the manufacturer.

### 2.9. Lipid Profiling

Lipid profile (total cholesterol (TC), triglycerides (TGs), low-density lipoprotein-cholesterol (LDL-C), and high-density lipoprotein-cholesterol (HDL-C)) was estimated by using enzymatic kits procured from Agappe Diagnostics Ltd. (Kerala, India).

### 2.10. Assessment of Oxidative Stress in Brain Tissue

#### 2.10.1. Estimation of Lipid Peroxidation Level

Malondialdehyde (MDA), a byproduct of lipid peroxidation, was measured in brain homogenate to detect lipid peroxidation (LPO) [24]. In brief, 0.1 mL of brain homogenate was mixed with 1.5 mL of acetic acid (20%, pH 3.5), 1.5 mL of TBA (0.8%), and 0.2 mL of sodium dodecyl sulfate (8.1%). This mixture was then incubated at 100 °C for 1 h. The mixture was then added to 5 mL of n-butanol: pyridine (15:1%, *v*/*v*) and 1 mL of distilled water before being centrifuged at 3000× *g* rpm for 10 min. The organic layer was removed, and a spectrophotometer (Shimadzu UV-1900) was used to detect absorbance at 532 nm. Using a molar extinction coefficient of 9.6 × 10^3^ M^−1^ cm^−1^, the amounts of MDA created in each sample were represented as nmol of MDA formed h-1 mg-1 of protein.

#### 2.10.2. Glutathione S-Transferase Activity Assay

The activity of glutathione S-transferase was measured by the method of Habig et al. [25]. The reaction mixture contained 3.0 mL of 0.1 M phosphate buffer (pH 7.4), 1.0 mM 1-chloro-2,4-dinitrobenzene, and 0.1 mL of PMS. The change in absorbance was measured (Shimadzu UV-1900) at 340 nm, and enzyme activity was calculated using the molar extinction value of 9.6 × 103 M^−1^ cm^−1^ as the number of CDNB conjugates generated per minute per mg.

#### 2.10.3. Superoxide Dismutase (SOD) Activity Assay

A conventional technique was used to measure SOD activity [26]. Briefly, to 0.1 mL of PMS (10%), 2.875 mL of Tris–HCl buffer (50 mM, pH 8.5) and 25 μL of pyrogallol (24 mM in 10 mM HCl) were added. The SOD activity was measured at 420 nm and expressed as units mg^−1^ protein. The enzyme activity that inhibits the autoxidation of pyrogallol by 50% is considered one unit of the enzyme.

#### 2.10.4. Catalase (CAT) Activity Assay

CAT activity was measured using the Claiborne assay [27]. Briefly, to 0.1 mL of PMS (10%), 1.9 mL of phosphate buffer (0.05 M, pH 7.0) and 1 mL of hydrogen peroxide (0.019 M) were added. At 240 nm, changes in absorbance were noted. Using a molar extinction coefficient of 43.6 M^−1^ cm^−1^, catalase activity was estimated as nmol H_2_O_2_ consumed min^−1^ mg^−1^ protein.

### 2.11. Proinflammatory Cytokine Analysis

Using commercially available ELISA kits (RayBiotech), we quantified neural proinflammatory cytokines such as tumor necrosis factor-alpha (TNF-) and interleukin-6 (IL-6) in accordance with the manufacturer’s recommendations.

### 2.12. Neuron-Specific Markers Analysis

To determine the mechanism of neuroprotective action, the expression analysis of neuron-specific markers such as brain-derived neurotrophic factor (BDNF) and nerve growth factor (NGF) was assessed using commercial ELISA kits (RayBiotech) in accordance with the producer’s guidelines.

### 2.13. Histopathological Examination of Brain and Pancreas

For histological analyses, brain and pancreas tissue were kept in 10% buffered formalin solution. Brain and pancreatic tissues were cut into thin slices, dried, and then embedded in paraffin after being fixed in 10% buffered formalin solution. At least four cross-sections of 3–4 μm thickness were cut from each sample and subjected to staining with hematoxylin and eosin (H&E). Tissue sections were mounted with DPX mountant after two xylene washes that lasted two minutes each. After that, the slides were examined using bright-field microscopy. The Olympus BX50 microscope system (Olympus, Tokyo, Japan) was used for taking microphotographs.

### 2.14. Statistical Analysis

The standard error of the mean (SEM) is used to express data. When there were more than two experimental groups, the statistical significance of the difference between the groups was determined using one-way ANOVA and the Kruskal–Wallis test, followed by Dunn’s tests. GraphPad InStat v3 (San Diego, CA, USA) was used for analyzing the results.

## 3. Results

### 3.1. Induction of Diabetes

**Figure 2:**
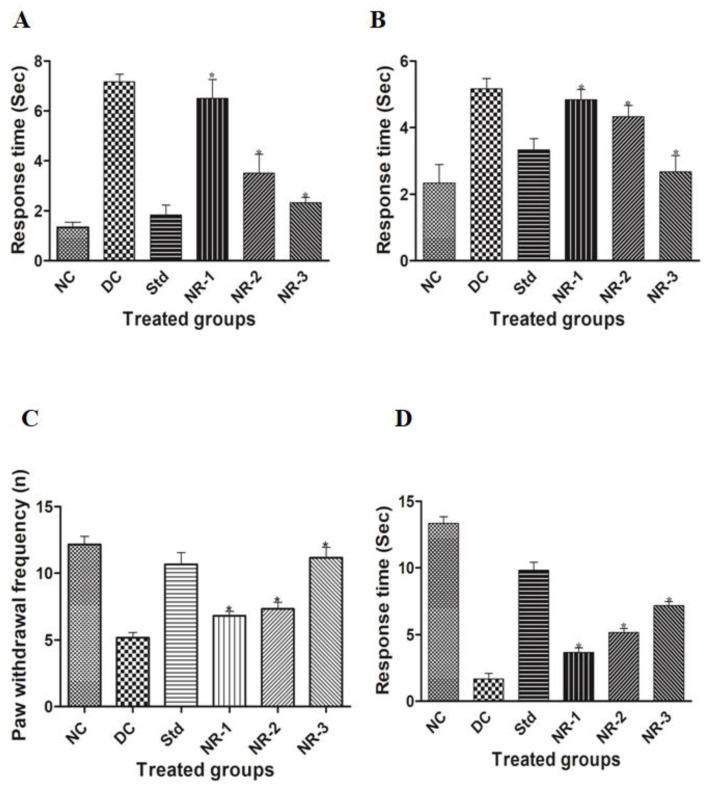
Effects of naringin on behavioral parameters: (**A**) acetone drop test, (**B**) plantar test, (**C**) hot plate test, and (**D**) tail flick test. Data are expressed as mean ± SEM (*n* = 6) and analyzed using one-way ANOVA and Kruskal–Wallis test, followed by Dunn’s test. NC, Normal control; DC, Diabetic control; Std, Standard; NR-1, Naringin 10 mg/kg bw. po; NR-2, Naringin 20 mg/kg bw. po; NR-3, Naringin 40 mg/kg bw. po (* *p* < 0.05).

HFD/STZ-induced diabetic rats developed diabetic neuropathy, which was confirmed by behavioral studies. Diabetic-neuropathy-induced animals showed a significantly delayed paw withdrawal tendency than normal rats in the acetone drop test, causing cold allodynia. The mean paw withdrawal tendency in normal rats was 1.33 ± 0.516, which increased to 7.16 ± 0.75 in diabetic neuropathy rats. NR administration to diabetic animals significantly (*p* < 0.05) attenuated the neuropathic pain in a dose-dependent manner. After 4 weeks of treatment of NR to neuropathic animals, the level of mean paw withdrawal tendency significantly lowered, 6.5 ± 1.87, 3.5 ± 1.87, and 2.33 ± 0.516 in NR-1, NR-2, and NR-3, respectively, in the treated groups of diabetic rats. Glibenclamide was used as a standard drug, and the mean paw withdrawal value was found to be 1.83 ± 0.98 (Figure 2A).

Similarly, the plantar test to assess the behavioral changes of neuropathic animals showed significant changes in the different NR-treated groups in a dose-dependent manner. In this experiment, the paw withdrawal response of the animals was noted in a duration-based manner. The mean paw withdrawal value of the diabetic group was found to be 5.16 ± 0.75 and 2.33 ± 1.36 in the case of normal rats. The glibenclamide-treated standard group mean paw withdrawal response was found to be 3.33 ± 0.816. The mean paw withdrawal response of the different treated groups of animals was evaluated as 4.83 ± 0.75, 4.33 ± 0.816, and 2.66 ± 1.21 in NR-1, NR-2, and NR-3, respectively (Figure 2B), which shows a significant reduction in paw withdrawal response.

In the case of the hot plate test or heat hyperalgesia, animals of each group were placed on a hot plate, the temperature of which was maintained at 55 ± 2 °C. The frequency of hind paw upliftment was measured at a 20 s cutoff time. The frequency of paw withdrawal in the case of diabetic animals significantly decreased (* *p* < 0.05) with respect to normal animals, 5.16 ± 0.98 and 12.16 ± 1.47, respectively. The treated animal group showed a significant dose-dependent response, i.e., 6.83 ± 0.75, 7.33 ± 1.21, and 11.16 ± 1.94 in NR-1, NR-2, and NR-3, respectively (Figure 2C).

In the case of the tail flick test, diabetic animals having a rapid tail flicking tendency were observed compared to the normal rats and standard-group rats. The mean tail withdrawal for diabetic rats was noted as 1.66 ± 1.03, whereas normal rats had a tail withdrawal time of 13.33 ± 1.21, and the standard group had a tail flicking time of 9.83 ± 1.47. The naringin-treated groups showed a dose-dependent ameliorating behavior of tail flicking tendency towards normal with an increase in the concentration of naringin, 3.66 ± 0.81, 5.16 ± 0.75 and 7.16 ± 0.75 in NR-1, NR-2, and NR-3, respectively (Figure 2D).

### 3.2. Serum Biochemical Estimation

**Figure 3:**
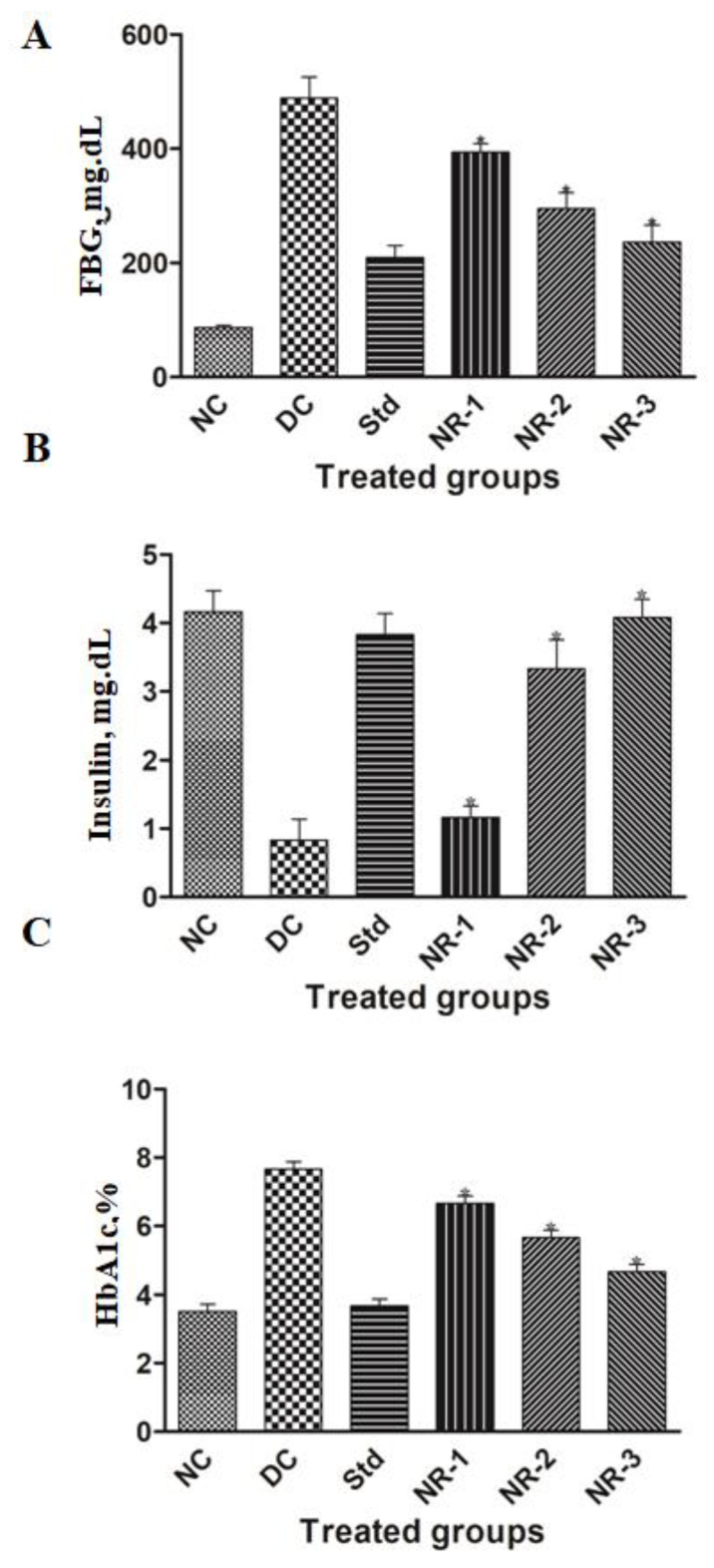
Estimation of serum biochemical levels in diabetic neuropathy rats and potential of naringin on (**A**) fasting blood glucose level (FBG) (**B**) insulin level, and (**C**) glycated hemoglobin % (HbA1c%). Data are expressed as the mean ± SEM (*n* = 6) and were analyzed using one-way ANOVA and Kruskal–Wallis test, followed by Dunn’s test. NC, Normal control; DC, Diabetic control; Std, Standard, NR-1, Naringin 10 mg/kg bw. po; NR-2, Naringin 20 mg/kg bw. po; NR-3, Naringin 40 mg/kg bw. po (* *p* < 0.05).

HFD/STZ successfully induced hyperglycemia in the experimental animals. Prolonged hyperglycemia caused the induction of diabetic neuropathy in the animals, as confirmed by behavioral studies. The mean FBG level of the animals in the diabetic group was found to be 488.16 ± 90.71 mg/dL, while normal animals had an FBG of 86.66 ± 9.11 mg/dL. A significant (*p* < 0.05) decrease in the mean FBG level was observed in the different groups of animals treated with different doses of naringin, i.e., 393.83 ± 38.11, 294.66 ± 69.05, and 236.83 ± 70.64 mg/dL in NR-1, NR-2, and NR-3, respectively (Figure 3A). The mean FBG level of the standard group of animals treated with the standard drug glibenclamide was found to be 209.16 ± 51.95 mg/dL.

Insulin is the key hormone known for glycemic regulation in our body. The mean insulin level of normal animals was found to be 4.16 ± 0.75 ng/mL, which decreased in the case of the diabetic groups to 0.83 ± 0.75 ng/mL. A dose-dependent increase was observed in the case of the NR-treated groups. The standard group of animals had a mean insulin level of 3.83 ± 0.75 ng/mL (Figure 3B). Similarly, the mean glycated hemoglobin level of diabetic animals was found to be 7.66 ± 0.51 compared to normal animals, which had a lower mean value, i.e., 3.5 ± 0.54, in the NR-treated groups. As we increased the dose of NR, a significant reduction in HbA1c% was observed. The mean HbA1c% of NR-1, NR-2, and NR-3 was found to be 6.66 ± 0.51, 5.66 ± 0.51, and 4.66 ± 0.51, respectively (Figure 3C).

### 3.3. Lipid Profile

**Figure 4:**
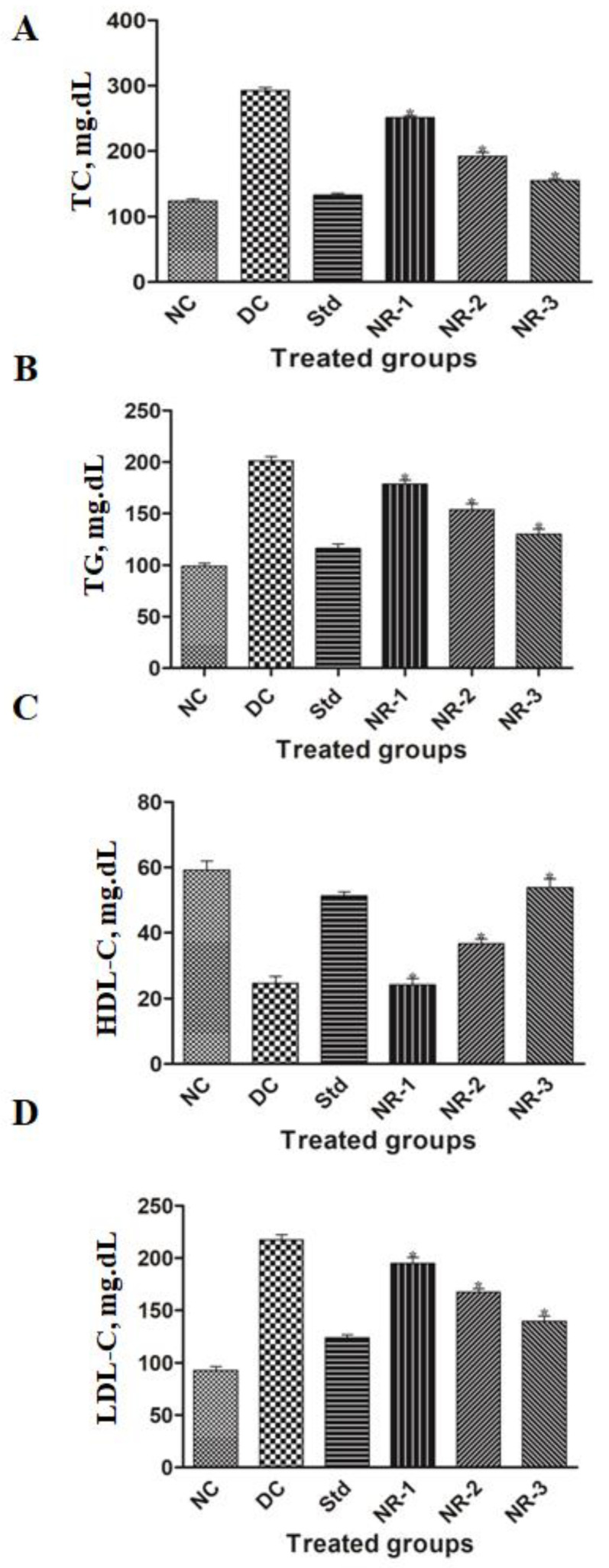
Estimation of lipid profile of diabetic neuropathy rats and potential of naringin on (**A**) total cholesterol (TC) level, (**B**) triglyceride (TG) level, (**C**) high-density lipid-cholesterol (HDL-C) level, and (**D**) low-density lipid-cholesterol (LDL-C). Data are expressed as the mean ± standard error of the mean (*n* = 6) and were analyzed using one-way ANOVA and Kruskal–Wallis test, followed by Dunn’s test. NC, Normal control; DC, Diabetic control; Std, Standard, NR-1, Naringin 10 mg/kg bw. po; NR-2, Naringin 20 mg/kg bw. po; NR-3, Naringin 40 mg/kg bw. po. (* *p* < 0.05).

The lipid profile of HFD/STZ-induced diabetic rats was greatly disturbed. STZ-induced diabetic rats had an elevated level of mean total cholesterol (TC) (292.66 ± 11.86) compared to normal rats (123.83 ± 7.33), whereas the NR-treated groups of rats showed 251.16 ± 7.27, 192.33 ± 13.66, and 154.66 ± 7.20, respectively, in the NR-1, NR-2, and NR-3 groups (Figure 4A) (* *p* < 0.05).

This result suggests that the mean cholesterol of the treated groups significantly (*p* < 0.05) ameliorates the total cholesterol level. NR showed hypoglycemic activity in a dose-dependent manner. The elevated level of triglycerides (TGs) (Figure 4B) and low-density lipid (LDL-C) (Figure 4D), and the decreased mean value of high-density lipid (HDL-C) (Figure 4C), were brought near about to normal in a dose-dependent manner. The maximum dose of NR showed the highest degree of amelioration in all the studied parameters.

### 3.4. Oxidative Stress

**Figure 5:**
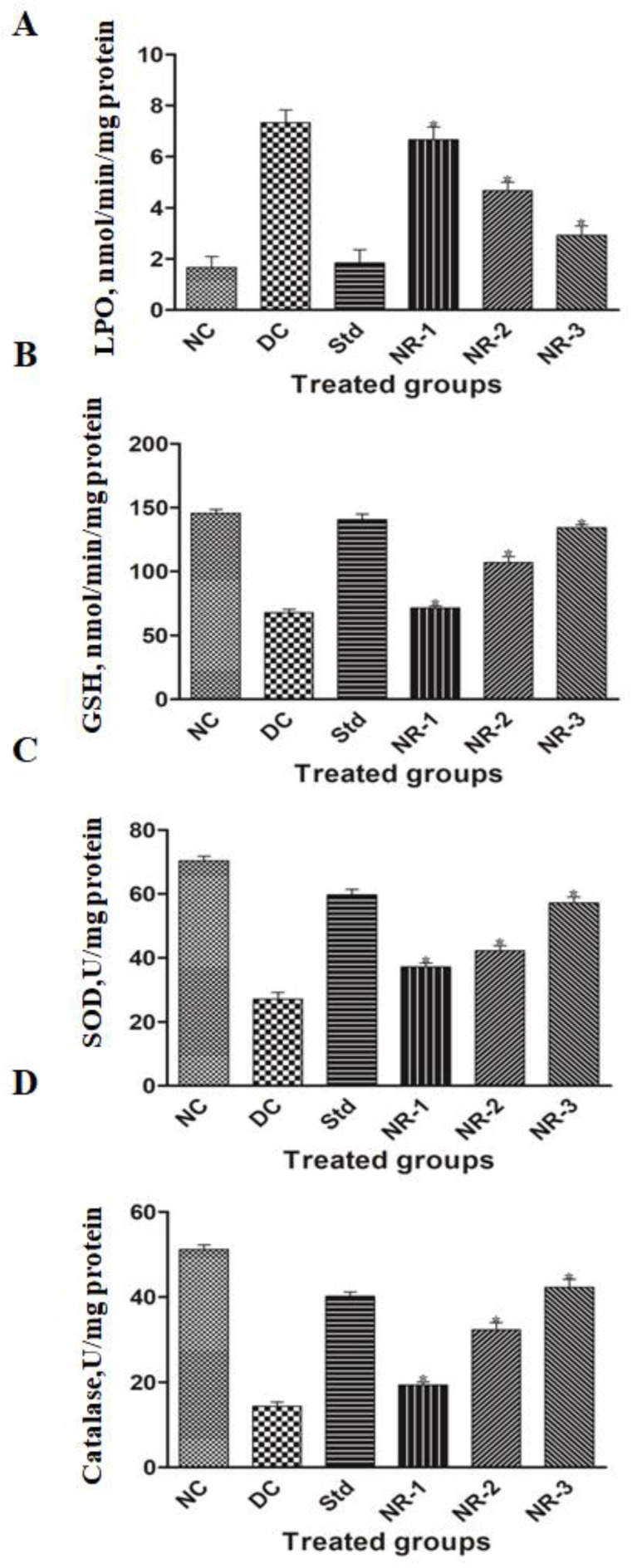
Estimation of oxidative stress in diabetic neuropathy rats and their attenuation by naringin on (**A**) lipid peroxidation (LPO) assay, (**B**) glutathione (GSH) activity assay, (**C**) SOD activity, and (**D**) catalase (CAT) activity. Data are expressed as the mean ± standard error of the mean (*n* = 6) and were analyzed using one-way ANOVA and Kruskal–Wallis test, followed by Dunn’s test. NC, Normal control; DC, Diabetic control; Std, Standard, NR-1, Naringin 10 mg/kg bw. po; NR-2, Naringin 20 mg/kg bw. po; NR-3, Naringin 40 mg/kg bw. po (* *p* < 0.05).

STZ-induced diabetes mellitus significantly reduced the mean antioxidant enzyme levels such as malondialdehyde((MDA) (Figure 5A), glutathione transferase (GST) (Figure 5B), superoxide dismutase (SOD) (Figure 5C), and catalase (CAT) (Figure 5D).

This causes an increase in the amount and activity of reactive oxygen species. NR significantly increases the level of the different antioxidant enzymes in a dose-dependent manner. The highest dose of NR had the highest degree of amelioration with respect to lower doses. The treated groups showed significant amelioration with respect to the diabetic group, while there was no significant difference observed between the treated groups and the maximum dose of NR with respect to the standard drug glibenclamide.

### 3.5. Proinflammatory Cytokine Analysis

**Figure 6:**
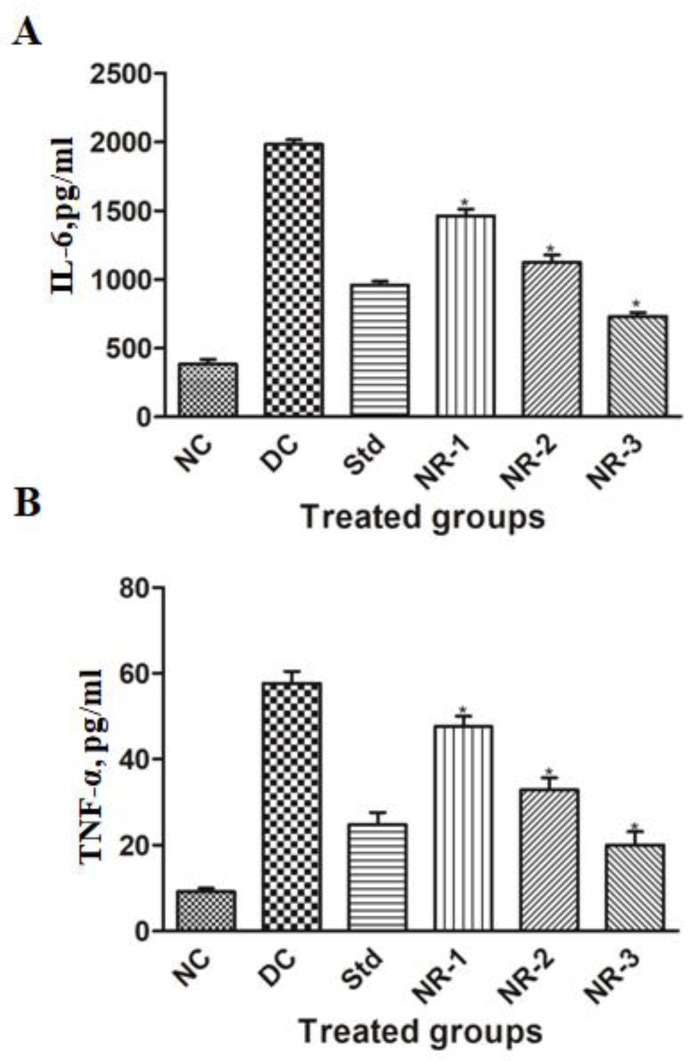
Effects of naringin on proinflammatory cytokines (**A**) IL-6 and (**B**) TNF-α protein expression. Data are expressed as the mean ± standard error of the mean (*n* = 6) and were analyzed using one-way ANOVA and Kruskal–Wallis test, followed by Dunn’s test. NC, Normal control; DC, Diabetic control; Std, Standard, NR-1, Naringin 10 mg/kg bw. po; NR-2, Naringin 20 mg/kg bw. po; NR-3, Naringin 40 mg/kg bw. po (* *p* < 0.05).

Proinflammatory cytokines are found to play an important role in the pathogenesis and progression of diabetic neuropathy. Our results demonstrate that the mean level of IL-6 and TNF-a increased significantly (*p* < 0.05) in diabetic neuropathic rats compared to normal rats. Animals treated with NR showed a significant reduction in the elevated level of IL-6 and TNF-α in a dose-dependent manner (Figure 6).

**Figure 7:**
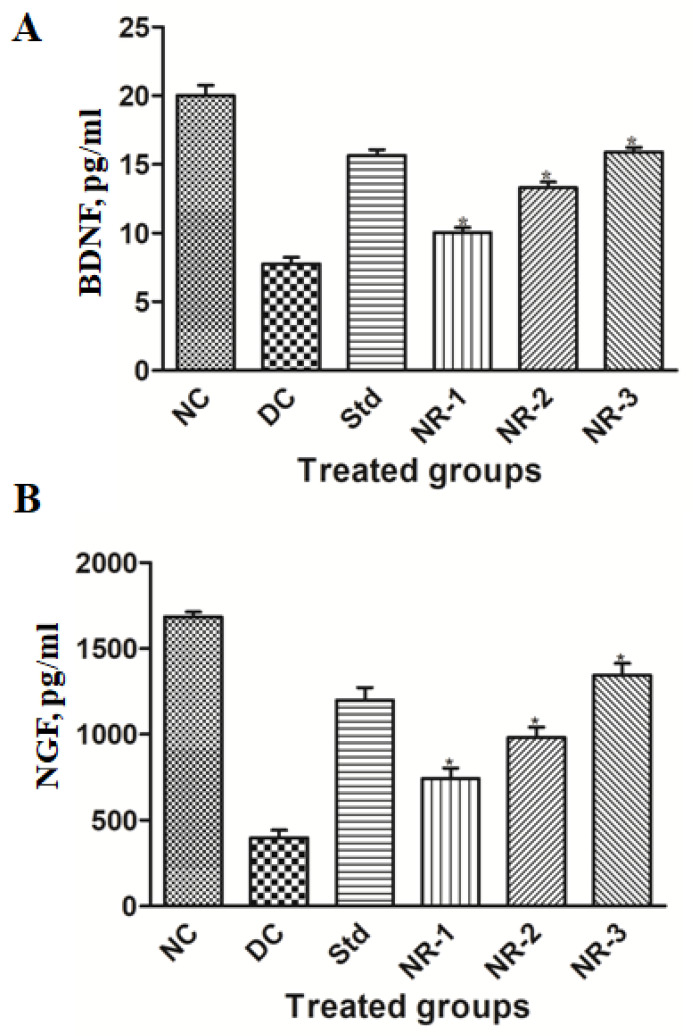
Effects of naringin on neuronal specific markers (**A**) BDNF and (**B**) NGF protein expression. Data are expressed as the mean ± standard error of the mean (*n* = 6) and were analyzed using one-way ANOVA and Kruskal–Wallis test, followed by Dunn’s test. NC, Normal control; DC, Diabetic control; Std, Standard, NR-1, Naringin 10 mg/kg bw. po; NR-2, Naringin 20 mg/kg bw. po; NR-3, Naringin 40 mg/kg bw. po (* *p* < 0.05).

Neurotrophins are proteins that consist of mainly NGF and BDNF responsible for increasing neurons and their damage prevention. In our study, we found that the mean level of NGF and BDNF of diabetic neuropathy animals decreased significantly (*p* < 0.05) compared to normal animals (Figure 7), while the treatment of NR showed the regeneration potential of NGF and BDNF in a dose-dependent manner.

### 3.6. Histopathology

**Figure 8:**
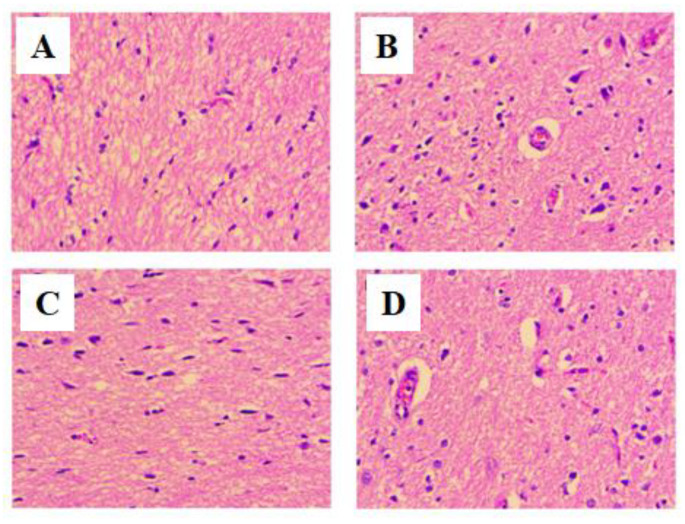
Representative hematoxylin and eosin (H&E) stained brain sections from neuropathic rats (*n* = 6) treated with (**A**) normal saline and diet (NC), (**B**) diabetic rats (DC), (**C**) rats treated with standard drug (Std), and (**D**) animal treated with highest dose of NR for a period of 4 weeks treatment (40× × 10× = 400×).

A simple and established H&E staining was used to determine the effect of NR-3 on brain architecture [28,29]. The H&E staining of the brain section of normal animals showed the regular architecture of the brain (Figure 8A). STZ-induced diabetic neuropathy rats showed altered structure in neurons, glial cells, and myelin sheath. Compared to normal animals, micrographs of diabetic neuropathy rats showed neuronal loss around the neurons and microglia neutrophils (Figure 8B). Treatment of DN rats with NR-3 showed a remarkable establishment of the usual architecture of the animal brain (Figure 8D).

**Figure 9:**
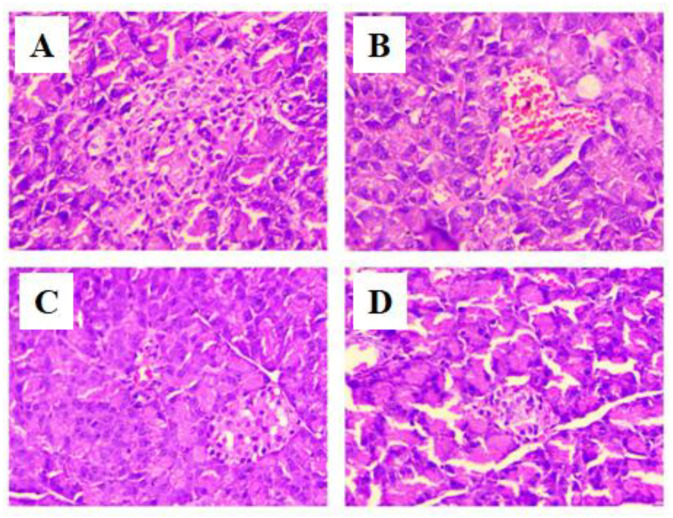
Representative hematoxylin and eosin (H&E) stained pancreas sections from neuropathic rats (*n* = 6) treated with (**A**) normal saline and diet (NC), (**B**) diabetic rats (DC), (**C**) rats treated with standard drug (Std), and (**D**). rats treated with highest dose of NR for a period of 4 weeks treatment (40× × 10× = 400×).

Histopathological analysis of the pancreas of the normal control (Figure 9A) rats showed the normal architecture of the pancreas containing alpha and beta cells in islets of Langerhans forming the endocrine portion compartmented by a conjunctive membrane. The outside of this region forms the exocrine portion consisting of acini. The cells of islets are polygonal with regular nuclei. STZ-induced diabetic neuropathy causes atrophy in islets and disturbs their normal architecture. The exocrine part shows the lymphocytic infiltrate between acini. Changes in the shape of acinar vacuolation and atrophy with pancreatic steatosis were observed (Figure 9B). Treatment of DN rats with NR-3 showed positive changes in the pancreatic structure (Figure 9D), while the standard group treated with glibenclamide showed the normal architecture of the pancreas (Figure 9C).

## 4. Discussion

STZ-induced diabetic neuropathy has the characteristics of allodynia and hyperalgesia due to elevated nociceptive response and reduced threshold to painful stimuli [30]. Animals also demonstrated clinicopathological traits such as biochemical, oxidative, and metabolic alterations in addition to these behavioral tests [31].

The acetone drop test, pinprick test, plantar test, and tail flick test are methods used for the record behavioral analysis of STZ-induced diabetic neuropathy animals [32,33]. Long-term diabetes persistence in animals results in sensitization of neurons due to damage to motor and sensory fibers leading to a reduction of paw withdrawal latency. The reduced pain threshold of normal animals and its dose-dependent amelioration were observed in naringin-treated animals. These results are similar to previous studies [34,35].

Hyperglycemia is the most prominent feature of diabetes mellitus leading to diabetic complications such as DN [34]. Studies have confirmed that hyperglycemia leads to glycation of hemoglobin (HbA1c) and an elevated level of HbA1c, which is a scale of advanced glycosylated end-products (AGEs) linked with delayed wound healing or leading to neuronal injury [36]. Treatment with naringin to DN animals significantly reduced the blood glucose level as well as HbA1c level in a dose-dependent manner.

Naringin reduces hypercholesteremia in rats fed a high-fat, high-cholesterol diet. It also inhibits HMG-CoA reductase and lowers plasma LDL and triglycerides without affecting the level of HDL cholesterol [37]. By this, we can say that naringin decreases the metabolic syndrome by overexpression of AMPK and underexpression of key enzymes for gluconeogenesis and reduces the activity of HMG-CoA reductase.

By acting as an antioxidant or free radical scavenger, phytochemicals enhance health [38]. ROS and free radicals are produced when the normal redox status of cells changes, which causes cytotoxicity and damages every component of the cell [39]. Naringin decreases lipid peroxidation and the buildup of ROS [40]. In HFD/STZ-induced diabetic rats with hyperglycemia, naringin boosts antioxidant defense system activity-induced oxidative stress by lowering SOD, GSH, and catalase activity [41]. MDA levels in DN rats were elevated compared to treated groups mainly responsible for dismantling lipid membranes by disturbing the double bond in the unsaturated fatty acids of the membrane, finally damaging the tissues. The elevated level of lipid peroxidation suggests increased oxidative stress, which was reduced by drug treatment. Vascular endothelial damage is predominantly caused by superoxide and hydroxyl radicals, which is protected by SOD by transforming them into H_2_O_2_ [42]. The treatment of naringin in DN rats significantly restored the altered levels of these antioxidant enzymes, which showed its antioxidant potential.

According to recent research, neuropathic pain and inflammation following nerve injury are strongly correlated [43]. A complicated illness known as neuropathic pain is brought on by numerous types of peripheral nerve damage, such as immunological and metabolic disorders, diabetes, infections, or drug-induced neuropathy [44]. Nearly all neuropathic pain types are caused by peripheral nerves. Peripheral nerves that respond to pain exhibit a remarkable level of flexibility in sensory neurons and the spinal cord [45]. Proinflammatory cytokines take part in this immune activation and direct the initial immune reaction. However, these inflammatory mediators have the potential to directly enhance neuronal excitability, harm myelin, and change the permeability of the blood–nerve barrier (BNB), all of which might cause edema and additional immune cell infiltration. Proinflammatory cytokines have frequently been linked to neuropathic pain induction, increased sensory afferent excitability, and demyelination and degeneration of peripheral neurons [43]. Inflammation is a complex biological process of vascular tissue to tissue injury, damaged cells, pathogens, or irritants, which leads to autoimmune diseases and can be controlled by using anti-inflammatory compounds having inflammatory responses [46]. Various preclinical and clinical studies have shown the pathogenic role of inflammation, particularly cytokine and chemokine production in DN. The activation of IKKβ/NF-κB has been implicated as a central player in the process of inflammation. A number of stimuli that promote type 2 DM, such as hyperglycemia, oxidative stress, and proinflammatory cytokines, activate NF-κB [47]. In turn, NF-κB upregulates the expression of proinflammatory genes such as IL-6 and TNF-α. NF-κB also promotes neuronal apoptosis and causes suppression of antioxidant gene activation by Nrf-2 downregulation, leading to the weakening of innate immune defense indirectly [48]. Overproduction of TNF-a in microvascular and neuronal tissues is the key feature of DN, which leads to microangiopathy and nerve damage [49]. Therefore, it is imperative to find suitable compounds and phytochemicals that can disrupt this perpetual pathway and can be used in combination therapy in treating chronic inflammation and diabetic complications. Here, we have explored the anti-inflammatory activity of naringin and found a dose-dependent reduction in TNF-a and IL-6 production by naringin, which are in line with previous studies [50].

Diabetic neuropathy may be prevented and/or treated using the regulatory functions of cytokines in nerve regeneration and degeneration [51]. Brain-derived neurotrophic factor (BDNF) is a neurotrophin that encourages neuronal survival, development, and neurogenesis [52]. A growing body of research has shown that BDNF also plays a pronociceptive role by dramatically promoting disinhibition of synapses at the spinal dorsal horn lamina I [53,54]. To stop the GABA effect of hyperpolarization, BDNF is produced and released by activated microglia P2X4 receptors [55]. Strong modulatory action exhibited by BDNF is well known, especially during brain plasticity events. BDNF has also been shown to perform a pronociceptive effect in peripheral neuropathic pain models due to the fact that it stimulates the potentiation of the NMDA receptor via the activation of TrkB and the phosphorylation of NMDAR-2B by Src-family kinase Fyn [54,56].

NGF is thought to be the most significant neurotrophic factor for diabetic neuropathy and linked to morphological variation, neuron regeneration, and the expression of neurotransmitters [57,58,59]. Expression of NGF in the nerves of STZ-induced rats decreases [60]. Additionally, research has revealed that the severity of sensory impairment is connected with the levels of NGF in the skin of individuals with diabetic neuropathy, which have been shown to be significantly lower [61,62]. Our findings are in accordance with the above-mentioned studies that the level of NGF decreases in STZ-induced diabetic neuropathy rats. These results could be explained by the high blood glucose level and insulin deficit being lowered by NGF expression in the target tissues, along with a decrease in NGF synthesis in Schwann cells. Additionally, reduced reverse axoplasmic transport and decreased affinity of the NGF receptor (TrkA) could both contribute to the downregulation of NGF expression [63,64]. Treatment with naringin dramatically reduced the elevated level of NGF in a dose-dependent manner.

Rats with chronic hyperglycemia suffer substantial brain damage in a variety of brain regions. By lowering oxidative stress, treatment with antioxidants containing naringin protected brain tissues from the damaging effects of DM. The findings of the current study suggest that DM may have positive effects on both preventing and healing brain injury. Numerous studies have demonstrated the connection between oxidative stress and cell loss/apoptosis, which is one of the main causes of pancreatic cell death [65]. Naringin boosted the expression of PPAR and PDX-1 and improved beta-cell regeneration to exert its antidiabetic effects. Beta-cell function is restored by PPAR, while pancreatic growth and beta-cell differentiation are controlled by PDX-1 [66]. These results have shown the potential of naringin to reduce diabetic complications by decreasing the level of proinflammatory cytokines, which are considered essential factors in promoting diabetic mellitus in general and diabetic neuropathy in particular. It will be interesting to discover the molecular pathways involved in reducing proinflammatory and inducing anti-inflammatory cytokines by naringin.

## 5. Conclusions

In conclusion, the result of various behavioral, biochemical, and molecular studies demonstrates the ameliorative properties of naringin against STZ-induced DN in rats through antihyperglycemic, antioxidative, and anti-inflammatory properties. Furthermore, behavioral and histological examinations of the brain and pancreas indicate that the damage caused by STZ-induced DN in rats was remarkably reduced following the administration of naringin in a dose-dependent manner. Therefore, naringin is a potential flavanone-bearing disease-modifying property to inhibit diabetes-induced neuropathic pain.

## Supplementary Materials

The following supporting information can be downloaded at: https://www.mdpi.com/article/10.3390/life12122111/s1, Figure S1: Schematic presentation of the course of T2DM inductions induced by STZ/HFD diet and the effect of naringin treatment.
