# Supplementary material for: Naringin Attenuates the Diabetic Neuropathy in STZ-Induced Type 2 Diabetic Wistar Rats
Source: Life (Basel). 2022 Dec 15;12(12):2111. doi: 10.3390/life12122111 (PMC9782177; doi:10.3390/life12122111)
Supplement: Supplementary file 1 [file life-12-02111-s001.zip › life-2026606-supplementary.pdf]

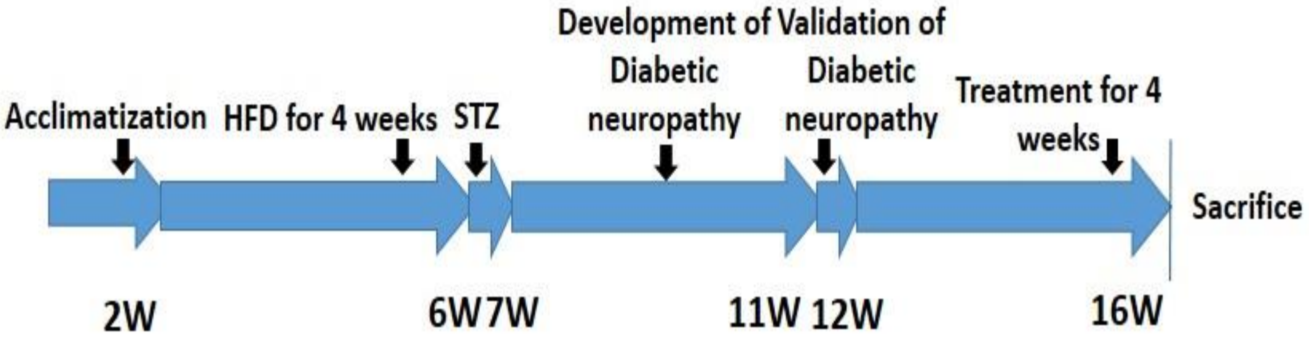

Figure S1. Schematic presentation of the course of T2DM inductions induced by STZ/HFD diet and the effect of naringin treatment.
